# Direct thyroid toxicity and sex differences in mitotane-induced hypothyroidism and dyslipidemia in thyroidology

**DOI:** 10.1016/j.clinsp.2026.101001

**Published:** 2026-05-28

**Authors:** Ilker Sengul, Demet Sengul

**Affiliations:** a Thyroidology Unit, Giresun University Faculty of Medicine, Giresun, Turkey; b Division of Endocrine Surgery, Giresun University Faculty of Medicine, Giresun, Turkey; c Department of General Surgery, Giresun University Faculty of Medicine, Giresun, Turkey; d Department of Pathology, Giresun University Faculty of Medicine, Giresun, Turkey

## Introduction

The management of Adrenocortical Carcinoma (ACC) remains a challenge, primarily due to the multifaceted toxicity profile of mitotane. We read with interest the recent valuable article by Tizianel et al.^1^ regarding mitotane-induced hypothyroidism and dyslipidemia. Their findings on sex-specific risks and direct thyroid cytotoxicity warrant further clinical and mechanistic discussion. This commentary is of multidisciplinary relevance to surgeons, endocrinologists, and oncologists, justifying its publication for the readership of *Clinics*.

## Mechanistic analysis (the “double-hit” hypothesis)

The investigators' use of the FRTL-5 rat thyroid cell model demonstrated a direct, dose-dependent cytotoxic insult to follicular cells, with an IC50 of approximately 50 µM (approx. 16 mg/L). Notably, this aligns with the upper limit of the human therapeutic window. This differential sensitivity may be attributable to tissue-specific expression of drug transporters, such as MDR1, or variations in intracellular drug metabolism, though this requires further investigation. When contrasted with the 80 µM threshold required to reduce pituitary cell viability in T-alpha-T1 models, these findings might suggest the thyroid gland is uniquely susceptible. Clinicians must recognize that “well-managed” patients at the high end of the therapeutic range (14–20 mg/L) are likely experiencing direct primary thyroid failure. Consequently, the clinical biochemical signature of low FT4 with an “inappropriately” low TSH should be reconsidered as a “double-hit” endocrinopathy: a synergistic suppression of the HPT axis coupled with a direct primary thyrotoxic insult.

### Sex-specific susceptibility

The authors’ identification of a distinct sexual dimorphism is salient. Females exhibit a substantially higher risk of central hypothyroidism (HR = 6.41). Conversely, males manifest dyslipidemia significantly earlier, with a median onset of 6-months compared to 20-months in females. Such differences likely reflect variations in drug clearance. This disparity necessitates a phenotype-stratified surveillance strategy.

### Actionable clinical protocols

We propose concrete monitoring protocols based on these sex-specific risks. Female patients should receive monthly FT4 monitoring during the first six months of therapy. Male patients require biweekly lipid profile assessments during dose escalation. Furthermore, the implementation of a standardized, stepwise escalation protocol ‒ prioritizing agents such as rosuvastatin or pravastatin due to mitotane-induced CYP3A4 induction, and incorporating ezetimibe or bempedoic acid ‒ is a necessary refinement.

## Conclusion

Tizianel et al.^1^ underscore the need for personalized endocrinological stewardship. Future investigations should focus on genomic variations in drug clearance to further refine personalized care in ACC management.

## Artificial intelligence (AI) or AI-assisted technologies

No generative AI or AI-assisted technologies have been used in the creation and production of this article.

## Data availability

The datasets used and/or analyzed during this study are available from the corresponding author upon reasonable request.

## Ethics approval

Not applicable.

## Authors' contributions

IS: Conceptualization; formal analysis; ınvestigation; methodology; project administration; writing-original draft; writing-review & editing.

DS: Conceptualization; formal analysis; ınvestigation; methodology; project administration; writing-original draft; writing-review & editing.

## Funding

None declared.

## Declaration of competing interest

The authors declare no conflicts of interest.
